# Some Aspects of Research in Medicine

**Published:** 1895-06-08

**Authors:** 


### Some Aspects of Research in Medicine.

Medicine is kept alive by "research." The jaded theologian looks and longs for the end of the world: the physical investigator, on the other hand, perceives that few things are yet known as they ought to be known, and is happy in the thought that he himself may plunge into the fray of original research, and emerge, perchance, with many spoils of conquest. But research—physical research—is no child's play. If it were, the whole world and all its contents would have been completely investigated long ago.

Botany and zoology have done their part in observing and recording the morphological and structural affinities of their subject matters. Physiology and medicine have to look much deeper, and especially is this the case with medicine, which must search deepest of all. Never can medicine be in any sense complete until she knows, not only the structure and function of every part of the human body, but until she understands and can explain the action and interaction of its every molecule.

One of the early tasks of the investigator is the taking of a just and intelligent measure of what has been accomplished by his predecessors. To discover things which have been already discovered is a waste of energy and temper. The intelligent and effective researcher is he who studies first of all "the story of the past," and then with the warp and woof of that story clear and distinct in his mind, sets to work to investigate and comprehend the life and activity of the things which are now living and working within sight of his own scientific eye.

The circumstance which most depresses the eager investigator is the slow evolution of results that bear fruit in practice. Much of the research of the present day seems to be like the barren fig tree in ancient Palestine, and is anathematised accordingly And yet this "slowness," which is so characteristic of modern research, is exactly paralleled by almost every process in Nature which is designed to produce great and lasting results. It takes twenty-five years for Nature to produce a full-grown man, and twice twenty-five for her to bring him to intellectual maturity. What sorry specimens many of her men are, moreover, when they are full-grown and have reached their intellectual ripeness! If Nature, with all her powers and resources, takes so long to accomplish what is often so little, what wonder is it that man, whose period of intellectual maturity is so brief, and whose resources for many investigations may almost be denoted by a cypher, should accomplish little, and oftentimes nothing at all?

Notwithstanding all this, the passion for investigation, when it once lays hold of a man, scarcely ever leaves him. As Sir William Hamilton believed, "If a man is born an investigator he will investigate; if he resolves not to investigate he will investigate; in any case he will investigate." Herein is the hope of the modern world. Medicine will go on investigating though she should seem to be always leaving off where she begun. She is not really walking in a circle after this fashion, notwithstanding the appearance of things from day to day. What we have to do is to take long views; supply ourselves with larger maps, as Lord Salisbury suggested. When we look back for no longer a period than merely half a century, it is not the smallness but the largeness of the results which compels our admiration. Half a century of modern medicine, if placed before an intelligent generation with graphic clearness and simplicity, would fill all mankind with wonder.

One plea we would enter in looking forward to the research of the future. It is that the scope of our investigations may be widened. At the present moment the world, for medical researchers, consists of men, horses, dogs, frogs, rabbits, goats, and guinea-pigs, with their microbes, of course, thrown in. If physiology and pathology were the only objects of our research this might be well. But is there no science of therapeutics? And does therapeutics owe nothing at all to the vegetable kingdom? Most of our substances which are serviceable in treatment have been brought in from the vegetable kingdom. Yet the greater part of the vegetable world, even of the vegetable world within our own islands, still lies outside the therapeutists' ken. Our science here is painfully narrow, injuriously small and poor. Will the day ever dawn when therapy will be a popular field of scientific inquiry, and when that part of organic nature which has already yielded us such generous fruits will be treated by the scientist with that inquiring respect which is so certainly and obviously its due?

